# A Unique Case of Eisenmenger Syndrome Treated With Interventional Surgery

**DOI:** 10.7759/cureus.110128

**Published:** 2026-06-02

**Authors:** Yusheng Long, Shuping Qin, Changhua Mo, Mengjie Wang

**Affiliations:** 1 Cardiovascular Medicine, The People's Hospital of Guangxi Zhuang Autonomous Region, Nanning, CHN; 2 Laboratory Medicine, The People’s Hospital of Guangxi Zhuang Autonomous Region, Nanning, CHN; 3 Cardiology, The People's Hospital of Guangxi Zhuang Autonomous Region, Nanning, CHN

**Keywords:** adult congenital heart disease, atrial septal defect (asd), eisenmenger syndrome, interventional procedure, pulmonary valve stenosis

## Abstract

Atrial septal defect (ASD) is a common congenital heart disease. According to previous views, once Eisenmenger syndrome develops, the opportunity for surgical treatment is lost. This article reports a case of ASD combined with pulmonary valve stenosis (PS) in a patient who had experienced right-to-left shunting through the ASD for 40 years. What distinguishes this patient from other Eisenmenger syndrome patients is the presence of severe PS. We attempted to first relieve the severe PS and then perform ASD closure. Ultimately, the patient benefited from surgical treatment.

## Introduction

Eisenmenger syndrome is a condition characterized by progressive and irreversible elevation of pulmonary vascular resistance due to various causes, ultimately converting an original left-to-right shunt into a right-to-left or bidirectional shunt [1,2]. Initially, a large intracardiac defect, such as an atrial septal defect, leads to left-to-right shunting of blood at the atrial level, resulting in chronically increased pulmonary blood flow, which in turn causes pulmonary vascular remodeling and progressively increased resistance, eventually leading to irreversible pulmonary hypertension. When the pulmonary circulation pressure exceeds the systemic circulation pressure, the shunt reverses to right-to-left, and patients begin to exhibit systemic cyanosis, hypoxemia, erythrocytosis, etc. If atrial septal defect closure is performed at this stage, the "decompressing" right-to-left shunt on which the right ventricle originally depends is blocked, leading to a sharp increase in right heart load and pulmonary artery pressure. Cyanosis and hypoxemia may not improve; instead, right heart failure may be accelerated and cause death.

According to the 2018 AHA/ACC Guideline for the Management of Adults With Congenital Heart Disease, closure of an atrial septal defect is not recommended (Class III: Harm) in adults with right-to-left shunt [3]. Similarly, the 2020 ESC Guidelines for the management of adult congenital heart disease explicitly state that closure of an ASD is not recommended (Class III C) in patients with Eisenmenger syndrome [4]. However, the situation is different when pulmonary valve stenosis is also present. In such cases, the elevated right atrial and right ventricular pressures may not originate from increased pulmonary artery pressure but rather from the pulmonary valve stenosis, which can mimic Eisenmenger syndrome. In fact, the elevation of right atrial and right ventricular pressures in this context is reversible and can be relieved by eliminating the pulmonary valve obstruction.

This case offers some insight: for patients with an atrial septal defect combined with pulmonary valve stenosis, even if a right-to-left shunt has developed, one can still attempt to address the clear underlying cause (i.e., severe pulmonary valve stenosis) first and then attempt atrial septal defect closure. It should not be assumed that the appearance of a right-to-left shunt means the surgical window has closed, leading only to conservative medical management.

## Case presentation

The patient is a 49-year-old male who was diagnosed with atrial septal defect and pulmonary valve stenosis at the age of 10 and subsequently developed Eisenmenger syndrome. He was considered to have missed the opportunity for surgery and did not receive standardized long-term treatment. He was admitted to the hospital due to severe dyspnea over the past month.

**Figure 1:**
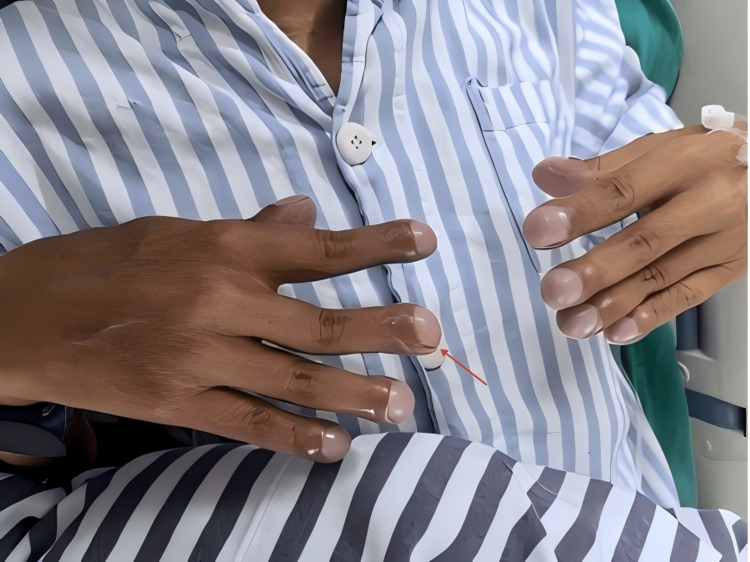
Typical clubbing finger caused by the patient's long-term chronic hypoxia

**Figure 2:**
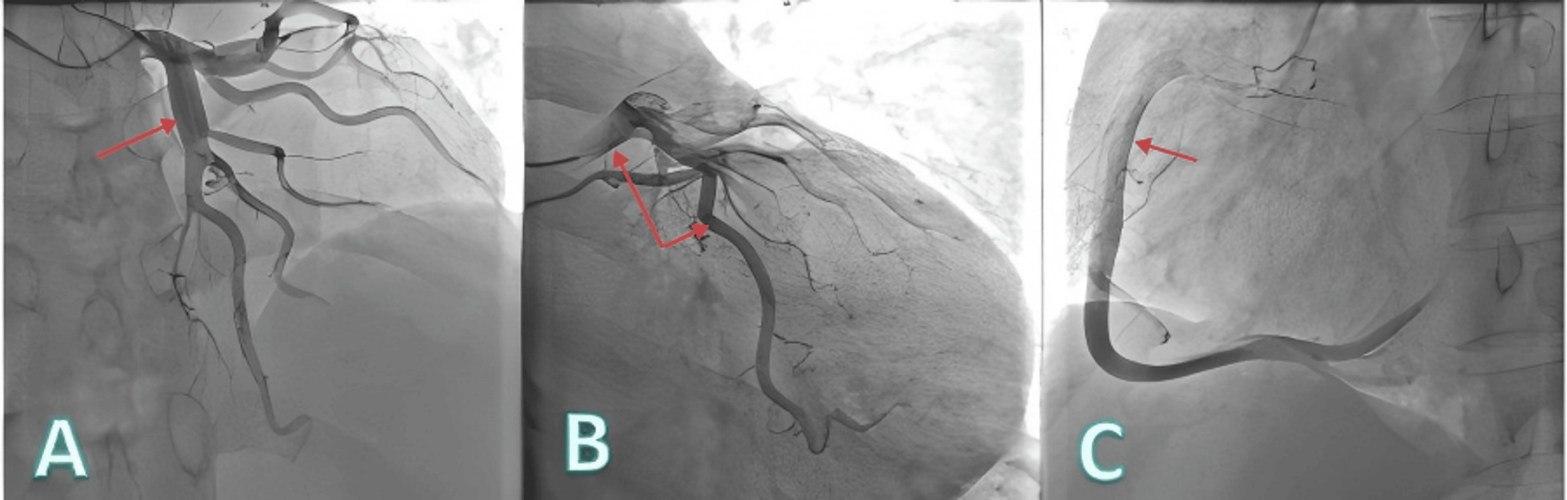
Using a 5F multipurpose angiography catheter, the images show thick and abundant coronary vessels A: The centered vessel is the left anterior descending artery. B: Centered vessels are the left main coronary artery and the left circumflex artery. C: The centered vessel is the right coronary artery.

**Figure 3:**
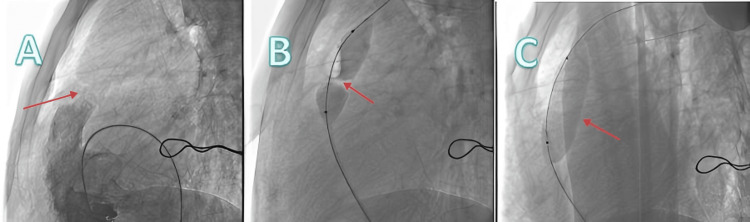
Pulmonary valve balloon valvuloplasty A: Right ventricular outflow tract angiography shows severe pulmonary valve stenosis, revealing a pulmonary valve opening of 5 mm, and measuring a pulmonary valve annulus diameter of 23 mm; B: The first pulmonary valve balloon dilation failed to open the stenotic valve (using a 25*50 mm pulmonary valve balloon); C: The second pulmonary valve dilation successfully opened the stenotic valve (using a 25*50 mm pulmonary valve balloon).

Upon admission, the patient could not lie flat and was unable to get out of bed because of breathlessness. Physical examination revealed cyanosis of the lips and extremities, mild pitting edema in both lower limbs, and significant clubbing of the fingers (Figure 1). Due to long-term hypoxia, hemoglobin was compensatorily elevated (225 g/L), and arterial blood gas oxygen partial pressure measured at an oxygen concentration of 37% was 43 mmHg (Table 1). Echocardiography indicated an atrial septal defect (secundum type), bidirectional shunt at the atrial level, and moderate pulmonary valve stenosis (Table 2). Coronary angiography revealed thick and abundant coronary vessels (Figure 2). The patient's low blood oxygen upon admission was considered related to the right-to-left shunt at the atrial level and reduced pulmonary blood flow caused by pulmonary valve stenosis. Initial treatment attempted to relieve the pulmonary obstruction by performing pulmonary valve balloon dilatation (Figure 3).

**Table 1:** Blood test and catheterization pressure measurement data

Indicator	Preoperative	One Month after Pulmonary Valve Dilation	Reference Range	Unit
Hemoglobin	225.00	169.00	130-175	g/L
Arterial Oxygen Partial Pressure	43.00 (Inhaled oxygen concentration: 37%.）	64.00 (No supplemental oxygen）	80-100	mmHg
Pulmonary Artery Pressure	16/12	14/7	15-30/4-14	mmHg
Right Ventricular Pressure	103/7	28/4	15-30/0-8	mmHg
Right Atrial Pressure	11	5	0-8	mmHg
Left Atrial Pressure	12	5	2-12	mmHg
Right Ventricular Outflow Tract Gradient	87	14	0-10	mmHg

**Table 2:** Ultrasound data

Measurement	Preoperative	One Month After Pulmonary Valve Dilation	After Pulmonary Valve Dilation and Atrial Septal Defect Closure	Reference Range	Unit
Left Atrial Anteroposterior Diameter	33	32	32	20 - 35	mm
Right Atrial Left - Right Diameter	57	40	43	25 - 35	mm
Left Ventricular End - Diastolic Anteroposterior Diameter	64	60	57	35 - 52	mm
Left Ventricular End - Systolic Anteroposterior Diameter	56	40	44	20 - 38	mm
Right Ventricular End - Diastolic Left - Right Diameter	36	40	38	20 - 35	mm
Interventricular Septal Thickness	13	12	12	7 - 11	mm
Left Ventricular Ejection Fraction	27.0	38.0	45.0	53 - 79	%
Pulmonary Valve Flow Velocity	3.8	2.6	2.5	—	m/s
Peak Pressure Gradient Across the Pulmonary Valve	59	27	25	—	mmHg
Tricuspid Regurgitation Area	8.2	3.9	3.9	—	mm²
Mitral Regurgitation Area	5.4	2.1	2.8	—	mm²

**Figure 4:**
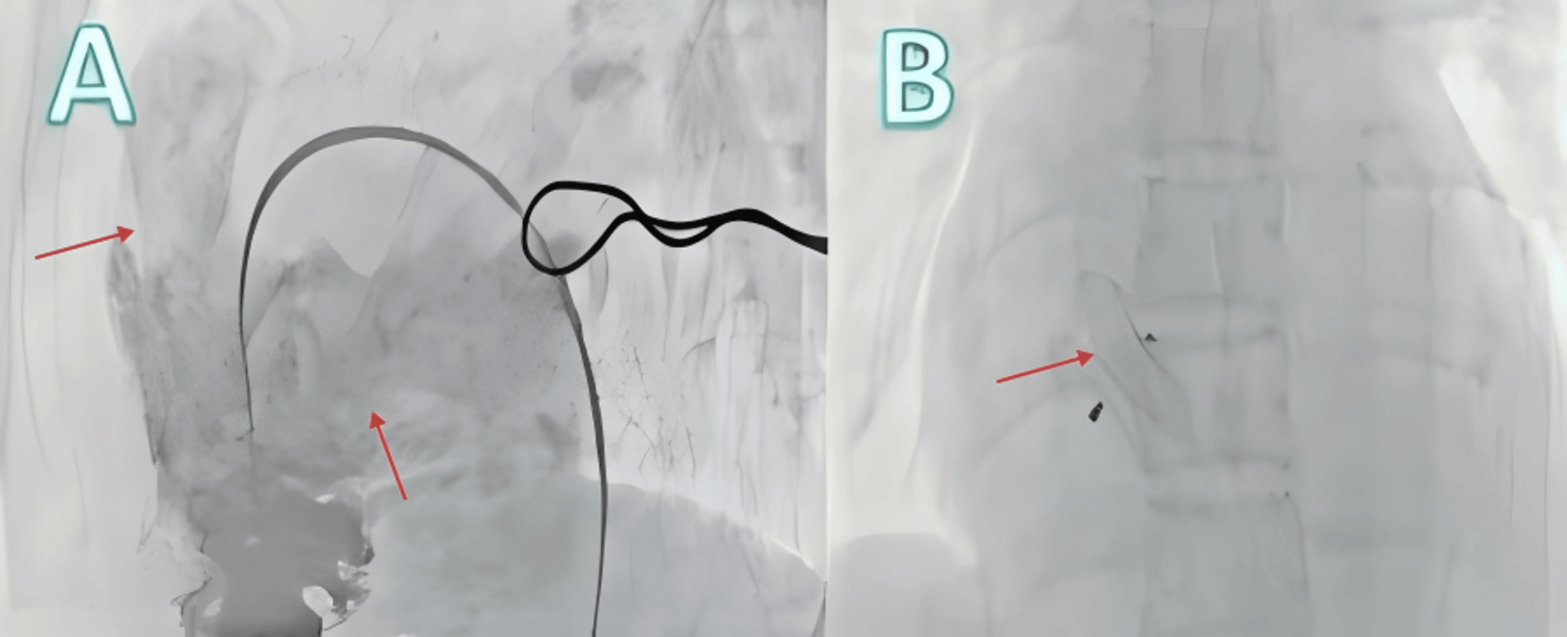
Atrial septal closure A: Shows the pulmonary valve after balloon dilation with no significant stenosis, and contrast agent flowing into the left atrium due to the atrial septal defect; B: Atrial septal defect occluder diameter 20 mm.

The patient returned for follow-up more than one month postoperatively. His dyspnea had significantly improved, with no severe dyspnea episodes during the past month. He was able to perform normal daily physical activities. Physical examination showed slightly improved cyanosis of the lips and extremities compared with before, and no edema in the lower limbs. Hemoglobin decreased to 169 g/L, and arterial oxygen partial pressure without oxygen supplementation increased to 64 mmHg. Repeat echocardiography showed that the degree of pulmonary valve stenosis had significantly improved compared with before, and mitral and tricuspid regurgitation were also markedly improved. Left ventricular ejection fraction had significantly increased compared with previous measurements, while pulmonary artery pressure showed no significant elevation. Therefore, an attempt was made to perform atrial septal defect closure (Figure 4). Postoperative echocardiography showed further improvement in left ventricular ejection fraction, and pulmonary artery pressure remained without significant elevation.

## Discussion

Eisenmenger syndrome is a condition characterized by progressive and irreversible elevation of pulmonary vascular resistance due to various causes, ultimately converting an original left-to-right shunt into a right-to-left or bidirectional shunt. The features of Eisenmenger syndrome include chronic hypoxemia and multiorgan involvement, manifesting as secondary erythrocytosis and secondary heart failure, among others, requiring multidisciplinary comprehensive treatment [5]. Once Eisenmenger syndrome has developed, the primary treatment focuses on medications to improve pulmonary arterial hypertension [6]. For high-risk patients who remain symptomatic despite poor response to drug therapy, some may opt for heart-lung transplantation [7], although this remains controversial [8].

The distinctive feature of this patient is that the pulmonary hypertension was not due to a true increase in pulmonary vascular resistance, but rather was caused by severe pulmonary valve stenosis. After relief of the pulmonary obstruction, one month of follow-up observation showed no significant increase in pulmonary vascular resistance. This suggests that in patients with atrial septal defect combined with pulmonary valve stenosis, even when right-to-left shunting has occurred, the shunt may be reversible, and attempting to relieve the pulmonary valve stenosis followed by atrial septal defect closure may still improve the patient's prognosis. As demonstrated in this patient, who had nearly 40 years of non-standardized treatment and exhibited clear signs of hypoxia, significantly decreased oxygen partial pressure, cyanosis, clubbing, markedly elevated hemoglobin, thick and abundant coronary vessels, and significantly reduced left ventricular ejection fraction, still benefited greatly from interventional procedures. Postoperatively, the patient could lie flat, engage in normal physical activity, and arterial oxygen partial pressure significantly increased, and left ventricular ejection fraction markedly improved.

Nevertheless, the potential risks must also be considered. The patient had severe pulmonary valve stenosis preoperatively, resulting in relatively low pulmonary blood flow, which might have masked the presence of pulmonary hypertension. After pulmonary valve dilation, pulmonary blood flow increased significantly, potentially unmasking underlying pulmonary hypertension. This is why, after the first pulmonary valve balloon dilation procedure, catheter pressure measurements showed higher pulmonary artery pressure than before (24/16 mmHg), and atrial septal closure was not performed immediately, due to concern that the pulmonary vasculature, after decades of "disuse," might not handle the increased flow. However, one month later, repeat catheterization showed that pulmonary artery pressure had actually decreased further (14/7 mmHg), leading to the decision to proceed with atrial septal closure. The overall outcome was satisfactory. After the two procedures, repeat echocardiography showed only mild residual pulmonary valve stenosis, significant improvement in left ventricular ejection fraction, and no indication of increased pulmonary artery pressure. With adjunct diuretic therapy, the degree of mitral and tricuspid regurgitation also improved compared to before.

## Conclusions

For patients with atrial septal defect combined with pulmonary valve stenosis, the right-to-left shunt may be reversible. If pulmonary artery pressure is not significantly elevated, attempting to relieve the pulmonary valve obstruction followed by atrial septal defect closure may be beneficial for the patient.
